# Supplementary material for: Oral Health Behaviour, Care Utilisation, and Barriers among Students with Disabilities: A Parental Perspective
Source: Healthcare (Basel). 2024 Sep 30;12(19):1955. doi: 10.3390/healthcare12191955 (PMC11475732; doi:10.3390/healthcare12191955)
Supplement: Supplementary file 1 [file healthcare-12-01955-s001.zip › healthcare-3197663-supplementary-1.pdf]

Parent Questionnaire

Thank you for taking part in this project. On completion of the questionnaire, please place it in the student's bag to be returned to the school later.

Student's Gender:

a) Male

b) Female

Student's Age: ....

Your relationship to the student:

a) Mother

b) Father

c) Others (specify)

Mother/Female Guardian's Education Level:

a) Illiterate

b) Elementary

c) High school

d) above high school

Father/Male Guardian's Education Level:

a) Illiterate

b) Elementary

c) High school

d) above high school

Maternal occupation

a) Employed

b) Unemployed

Paternal occupation:

a) Employed

b) Unemployed

Student's education level:

a) Elementary

b) b) Secondary

c) c) High school

Hand coordination and ability to brush own teeth

a) None

b) Severely limited

c) Able to do with some limitations

d) Able to do with no limitation (normal)

Tooth brushing:

a) Twice a day or more

b) Once a day

c) Irregular

d) Never

Dental flossing:

a) Yes

b) No

Sugar/sweets intake:

a) Never

b) Rarely in small quantity

c) Once or twice a day in small quantity

d) Once or twice a day in large quantity

e) More than twice a day in large quantity

Student's Oral Health Status:

a) Excellent

b) Good

c) Moderate

d) Poor

How often does the student visit the dentist per year?

a) Three or more times a year

b) Twice a year

c) Once a year

d) Emergency basis only

e) Never

If known, what type of dental procedures has the student received in the past? (Circle all that apply)

a) Cleaning teeth

b) Filling tooth/teeth

c) Oral examination

d) Crown/ Bridge

e) Denture (partial and or complete)

f) Root canal therapy

g) Tooth extraction

h) Emergency treatment (for broken tooth, pain etc.)

i) Unknown

Has the student received sedation for dental treatment in the past?

a) Yes

b) No

If the student has received sedation for dental treatment, what type? (Circle all that apply)

a) General anesthesia in a hospital setting

b) IV conscious in a hospital or clinic setting

c) Oral medication for sedation only in a hospital or clinic setting

d) Minimal inhaled sedation "laughing gas"

e) Not sure/do not remember

What type of facility does the student go to for dental care?

a) Hospital

b) Primary Health Center

c) Private general practice

d) Pediatric dental practice

Circle the level of difficulty for each of the following barriers to the student's dental care on a scale of 1–10: (1 indicates easy with No difficulty; 10 indicates extreme difficulty)

a) Finances (1-10): (Easy) 1 2 3 4 5 6 7 8 9 10 (Difficult)

b) Transportation (1-10): (Easy) 1 2 3 4 5 6 7 8 9 10 (Difficult)

c) Finding a dentist willing to treat (1-10): (Easy) 1 2 3 4 5 6 7 8 9 10 (Difficult)

d) Wait time for appointment (1-10): (Easy) 1 2 3 4 5 6 7 8 9 10 (Difficult)

e) Distance traveled (1-10): (Easy) 1 2 3 4 5 6 7 8 9 10 (Difficult)

f) Interior design of building (1-10): (Easy) 1 2 3 4 5 6 7 8 9 10 (Difficult)

g) Designated parking space (1-10): (Easy) 1 2 3 4 5 6 7 8 9 10 (Difficult)

h) Fear towards dental treatment (1-10): (Easy) 1 2 3 4 5 6 7 8 9 10 (Difficult)

i) Other -please list (------------) (1-10) (Easy) 1 2 3 4 5 6 7 8 9 10 (Difficult)

On a scale of 1–10, how satisfied are you with the student's current dental care?
(1 = extremely dissatisfied; 10 = very satisfied)

(Unsatisfied) 1 2 3 4 5 6 7 8 9 10 (very satisfied)

On a scale of 1–10, how important is it for the student to have access to routine dental care (e.g., exams and cleanings every 6 months)?
(1 = not important; 10 = extremely important)

(Not important) 1 2 3 4 5 6 7 8 9 10 (very important)

……………………………………………………………………………………………………

Thank you for your participation
